# Editorial: Fruit Responses to Biotic and Abiotic Stressors During Postharvest

**DOI:** 10.3389/fpls.2022.914841

**Published:** 2022-04-28

**Authors:** Isabel Lara, Claudia A. Bustamante, Natalia M. Villarreal

**Affiliations:** ^1^Postharvest Unit, AGROTÈCNIO-CERCA Center, Universitat de Lleida, Lleida, Spain; ^2^Bases bioquímicas de la calidad de frutos y exporte de fotoasimilados, Centro de Estudios Fotosintéticos y Bioquímicos (CEFOBI), Rosario, Argentina; ^3^Laboratorio de Bioquímica y Fisiología de la Maduración de Frutos, Instituto Tecnológico de Chascomús (INTECH, CONICET-UNSAM), Chascomús, Pcia, Buenos Aires, Argentina; ^4^Escuela de Bio y Nanotecnologías (EByN, UNSAM), Buenos Aires, Argentina

**Keywords:** fruit quality, postharvest, ripening, senescence, shelf life, stress

## Introduction

The ripening process encompasses many molecular, biochemical and physiological alterations in the course of which fleshy fruits acquire those sensory attributes which render them edible (Seymour et al., 2013; Kapoor et al., 2022). These modifications do not stop after harvest, and hence these produce are highly perishable commodities. Because postharvest deterioration causes important economic losses, a variety of postharvest strategies are applied in an attempt to preserve quality attributes and thus to extend storage and shelf life potential of fruits as well as other high-value horticultural produce such as cut flowers. Postharvest management needs to be tailored to each particular species, but very often involves refrigerated storage which can result in chilling injury and thus be detrimental for consumer acceptance. Other approaches for the postharvest management of horticultural commodities may also have negative effects on some quality traits and, at any rate, are not applicable in all cases.

This Research Topic was launched to gather novel information on responses to stress factors encountered during postharvest handling of horticultural produce, potentially providing hints for improved preservation of quality and marketability. The articles included in this Research Topic (a) examined the biochemical and molecular mechanisms involved in those responses, and (b) explored the efficacy of plant hormone treatments for the alleviation of stress symptoms. These studies considered a range of climacteric (apple, banana, peach, tomato) and non-climacteric (carambola, orange, pomegranate) fruit species as well as cut roses as an example of a commercially relevant floricultural crop, and are briefly presented below.

## Chilling Stress and Chilling Injury

The articles in this Research Topic illustrate the detrimental effects of cold storage on certain quality traits in some commercially important fruit crops, especially external appearance. For instance, bitter pit is an important disorder in apple affecting both fruit appearance and taste, occurring primarily during cold storage. Whereas generally considered to arise from low or unbalanced calcium levels, there is little information on the cell structure alterations involved therein. Qiu et al. observed increased amounts of amyloplasts in flesh cells of bitter pit-affected apples, and suggested this factor may disrupt calcium distribution leading to programmed cell death, imbalanced cell metabolism and bitter pit development.

Transcriptomic approaches allowed the identification of genes expressed differentially in response to chilling stress. Huang et al. identified 111 cuticle-related genes differentially regulated by chilling injury-inducing temperature in bananas, indicating a role for cuticle in chilling injury responses. Tomato fruit can also develop chilling injury symptoms upon transfer to ambient temperature, including accelerated softening and mealiness development. While such symptoms have been related to modified cell wall metabolism, results of transcriptome analysis undertaken by Hunter et al. suggest that accelerated softening was associated rather to turgor reduction. Chilling exposure also altered the expression of genes involved in protection and repair of oxidative damage, heat shock proteins and chaperonins, which could be used as molecular markers of cold response. Furthermore, epigenetic factors such as DNA methylation were apparently more relevant than gene expression for explaining differences associated to the development of mealiness in peach fruit kept under cold storage (Rothkegel et al.), and might also prove useful tools in breeding programs.

Non-climacteric fruits are also prone to chilling injury. In pomegranate, symptoms often manifest as peel browning, which could be delayed and attenuated by packaging in modified atmosphere, with or without 1-methylcyclopropene treatment (Valdenegro-Espinoza et al.). Contrarily, exogenous ethylene boosted lipid peroxidation and oxidative damage. Accordingly, 2,4-epibrassinolide applications to carambola fruit enhanced the antioxidant system, inhibited respiration rates and delayed senescence (Zhu et al.), in agreement with reports that brassinosteroids help counteracting both abiotic and biotic stress in plants (Kang and Guo, 2011).

## Other Stress Factors

Other studies compiled in this Research Topic addressed additional abiotic stress factors such as low O_2_ concentrations in storage atmosphere. Postharvest storage under controlled or modified atmospheres has been the object of intense research efforts, and it is a widespread practice for the mid- to long-term postharvest preservation of some temperate fruit crops. While the reduction in respiration rates generally delays ripening- and senescence-associated changes, the concomitant decrease in oxidative phosphorylation levels causes a shift to fermentative metabolism which may lead to detrimental effects on flavor and to some physiological disorders, thus compromising sensory quality and economic returns. Even so, considerably less published research on this topic is available for tropical fruits. This information was reviewed by Benkeblia. This survey indicated that low O_2_ levels can help extend the shelf life of tropical fruits, but also emphasised the wide range of metabolic processes affected, and the need to tailor these procedures on a case-by-case basis, since tropical fruits are remarkably diverse in structure, physiology and tolerance to O_2_ deprivation.

Water stress is also a relevant driver of fruit quality losses. Sharp increases in relative humidity during postharvest handling often result in non-chilling peel pitting (NCPP), an important peel disorder in citrus fruit. Romero et al. found that transcriptomic responses to abrupt rehydration in NCPP-affected oranges involve membrane disorganization, cell wall modification and proteolysis.

Horticultural produce is also sensitive to wound stress induced during handling and transportation. This is particularly relevant for ornamental commodities such as flowers, which will rapidly deteriorate in response to the outburst in ethylene production caused by mechanical damage. WRKY transcription factors (TFs) are involved in plant stress, senescence and wounding responses, and are known to interact with a range of plant growth regulators (Jiang et al., 2017). Accordingly, Jing et al. examined the events involved in perception and transmission of the wound signal in cut roses. Transcriptomic analysis in response to wound, exogenous ethylene, and a combination of both, allowed the identification of many genes expressed differentially, among which *RhWRKY33*, highly expressed in senescing, wounded and ethylene-treated petals. Silencing of *RhWRKY33* delayed senescence, which suggests a role as a positive regulator of ethylene-mediated wound responses.

## Conclusions

In summary, the articles in this Research Topic (a) identified differential patterns of gene expression and epigenetic regulation in response to a range of abiotic stress factors, (b) hinted at their potential as molecular markers of stress tolerance, and (c) evaluated the feasibility of plant hormone treatments for symptom relief. This Research Topic also highlighted the importance of fruit skin and overall appearance to meet consumers' expectations and ensure commercial revenues.

## Author Contributions

All authors listed have made a substantial, direct, and intellectual contribution to the work and approved it for publication.

## Funding

Current work at IL's lab was funded by Grant 2017 SGR 1108 (Generalitat de Catalunya, Catalonia, Spain), CAB's lab was funded by grants from Universidad Nacional de Rosario (80020180300050UR), and NV's lab received grants from ANPCyT (PICT-2018-3412 and PICT-2018-3166).

## Conflict of Interest

The authors declare that the research was conducted in the absence of any commercial or financial relationships that could be construed as a potential conflict of interest.

## Publisher's Note

All claims expressed in this article are solely those of the authors and do not necessarily represent those of their affiliated organizations, or those of the publisher, the editors and the reviewers. Any product that may be evaluated in this article, or claim that may be made by its manufacturer, is not guaranteed or endorsed by the publisher.
